# Plasma proteome association with coronary heart disease and carotid intima media thickness: results from the KORA F4 study

**DOI:** 10.1186/s12933-024-02274-3

**Published:** 2024-05-29

**Authors:** Mohamed A. Elhadad, Mónica del C. Gómez-Alonso, Chien-Wei Chen, Sonja Neumeyer, Thomas Delerue, Wolfgang Rathmann, Michael Näbauer, Christa Meisinger, Stefan Kääb, Jochen Seissler, Johannes Graumann, Wolfgang Koenig, Karsten Suhre, Christian Gieger, Uwe Völker, Annette Peters, Elke Hammer, Melanie Waldenberger

**Affiliations:** 1 Research Unit Molecular Epidemiology, Helmholtz Zentrum München, German Research Center for Environmental Health, Ingolstaedter Landstr. 1, 85764 Neuherberg, Germany; 2 Institute of Epidemiology, Helmholtz Zentrum München, German Research Center for Environmental Health, Neuherberg, Germany; 3 Department of Internal Medicine B, University Medicine Greifswald, Ferdinand-Sauerbruch-Str., 17475 Greifswald, Germany; 4 DZHK (German Center for Cardiovascular Research), partner site Greifswald, Greifswald, Germany; 5 Institute of Medical Information Sciences, Biometry and Epidemiology Medical Faculty, Ludwig-Maximilians-University, Munich, Germany; 6 German Center for Diabetes Research (DZD), Partner Site Düsseldorf, Düsseldorf, Germany; 7 Institute for Biometrics and Epidemiology, German Diabetes Center, Leibniz Center for Diabetes Research at Heinrich Heine University Düsseldorf, Düsseldorf, Germany; 8 Medizinische Klinik und Poliklinik I, Klinikum der Universität München, Munich, Germany; 9 German Research Center for Cardiovascular Disease (DZHK), Partner site Munich Heart Alliance, Munich, Germany; 10 Chair of Epidemiology, University of Augsburg, 86156 Augsburg, Germany; 11 Department of Cardiology, Medical Policlinic and University Clinic I, Munich, Germany; 12 Department of Internal Medicine IV, University Hospital of Ludwig-Maximilians-University, Munich, Germany; 13 Department of Medicine, Institute of Translational Proteomics, Philipps-Universität Marburg, Marburg, Germany; 14 Deutsches Herzzentrum München, Technical University Munich, Munich, Germany; 15 Institute of Epidemiology and Medical Biometry, University of Ulm, Ulm, Germany; 16 Bioinformatics Core, Weill Cornell Medicine-Qatar, Education City, 24144 Doha, Qatar; 17 Department of Biophysics and Physiology, Weill Cornell Medicine, 10065 New York, NY USA; 18 Interfaculty Institute for Genetics and Functional Genomics, University Medicine Greifswald, Greifswald, Germany

**Keywords:** Galectin-4, NCK1, Coronary artery disease, Stroke, Carotid intima media thickness, Atherosclerosis, Cardiovascular disease, Proteomics

## Abstract

**Background and aims:**

Atherosclerosis is the main cause of stroke and coronary heart disease (CHD), both leading mortality causes worldwide. Proteomics, as a high-throughput method, could provide helpful insights into the pathological mechanisms underlying atherosclerosis. In this study, we characterized the associations of plasma protein levels with CHD and with carotid intima-media thickness (CIMT), as a surrogate measure of atherosclerosis.

**Methods:**

The discovery phase included 1000 participants from the KORA F4 study, whose plasma protein levels were quantified using the aptamer-based SOMAscan proteomics platform. We evaluated the associations of plasma protein levels with CHD using logistic regression, and with CIMT using linear regression. For both outcomes we applied two models: an age-sex adjusted model, and a model additionally adjusted for body mass index, smoking status, physical activity, diabetes status, hypertension status, low density lipoprotein, high density lipoprotein, and triglyceride levels (fully-adjusted model). The replication phase included a matched case-control sample from the independent KORA F3 study, using ELISA-based measurements of galectin-4. Pathway analysis was performed with nominally associated proteins (p-value < 0.05) from the fully-adjusted model.

**Results:**

In the KORA F4 sample, after Bonferroni correction, we found CHD to be associated with five proteins using the age-sex adjusted model: galectin-4 (LGALS4), renin (REN), cathepsin H (CTSH), and coagulation factors X and Xa (F10). The fully-adjusted model yielded only the positive association of galectin-4 (OR = 1.58, 95% CI = 1.30–1.93), which was successfully replicated in the KORA F3 sample (OR = 1.40, 95% CI = 1.09–1.88). For CIMT, we found four proteins to be associated using the age-sex adjusted model namely: cytoplasmic protein NCK1 (NCK1), insulin-like growth factor-binding protein 2 (IGFBP2), growth hormone receptor (GHR), and GDNF family receptor alpha-1 (GFRA1). After assessing the fully-adjusted model, only NCK1 remained significant (β = 0.017, p-value = 1.39e-06). Upstream regulators of galectin-4 and NCK1 identified from pathway analysis were predicted to be involved in inflammation pathways.

**Conclusions:**

Our proteome-wide association study identified galectin-4 to be associated with CHD and NCK1 to be associated with CIMT. Inflammatory pathways underlying the identified associations highlight the importance of inflammation in the development and progression of CHD.

**Supplementary Information:**

The online version contains supplementary material available at 10.1186/s12933-024-02274-3.

## Introduction

Atherosclerosis, which is a cornerstone pathophysiological process of multiple disease forms including coronary heart disease (CHD) and stroke, is the leading cause of mortality worldwide [1]. Epidemiological studies of CHD and stroke have successfully identified major metabolic risk factors such as obesity, diabetes, dyslipidemia, and hypertension, which in turn helped to develop preventive strategies and to identify new drug targets [2].

Atherosclerosis is characterized by thickening of both the media and intima of the arterial wall, followed by plaque formation. Where this process impedes the proper delivery of oxygen-rich blood to the heart, it results in the development of CHD [3]. Specific mechanisms of atherosclerosis involve endothelial dysfunction, lipid accumulation, inflammation, oxidative stress, angiogenesis, matrix degradation, and thrombosis [3]. A marker of early atherosclerosis is carotid intima media thickness (CIMT), an ultrasound-based measurement of arterial wall thickness [4]. CIMT is clinically used to predict risk of atherosclerotic diseases like CHD and stroke [4–9]. An increase in CIMT of 0.1 mm has been found to be associated with 15% higher risk for myocardial infarction and 18% higher risk for stroke [10].

Identifying proteins linked to specific atherogenic-mechanisms sheds light onto the molecular pathophysiology of cardiometabolic disturbances. Although there have been multiple studies that found highly expressed proteins within atherosclerotic tissue, only a small number of these findings was replicated when using blood samples [11–13].

Proteomics can provide valuable clues to the underlying pathological mechanisms at the molecular level. The plasma proteome has been extensively characterized in cardiovascular disease, because of its availability and proximity to the cardiovascular system. Several studies applying mass spectrometry to quantify plasma proteins in small sample numbers have identified proteins associated with CHD [14–20]. A study using an aptamer-based platform, capable of profiling more than thousand analytes in plasma, detected many protein changes in the context of myocardial injury in a cohort of the Framingham Heart Studies, thus underscoring the potential of proteomics tools when applied to large human cohorts [21].

In the present study we aim to expand our knowledge of CHD pathophysiology, using quantitative data for 1129 proteins in plasma samples from 1000 individuals of the population-based KORA F4 study from a high-throughput aptamer-based analysis [23]. We explore the association of plasma protein levels with both CHD and CIMT, and additionally validate our findings in an independent sample from the KORA F3 cohort. We further explore possible roles of the associated proteins in the pathogenesis of atherosclerosis and CHD using pathway analysis.

## Materials and methods

### Study design of the discovery cohort

The KORA (Cooperative health research in the Region of Augsburg) study is an independent population-based cohort study in southern Germany [22]. The study was approved by the ethics committee of the Bavarian Medical Association and was carried out in accordance with the principles of the Declaration of Helsinki. All study participants signed written informed consent prior to their participation in the study.

**Fig. 1:**
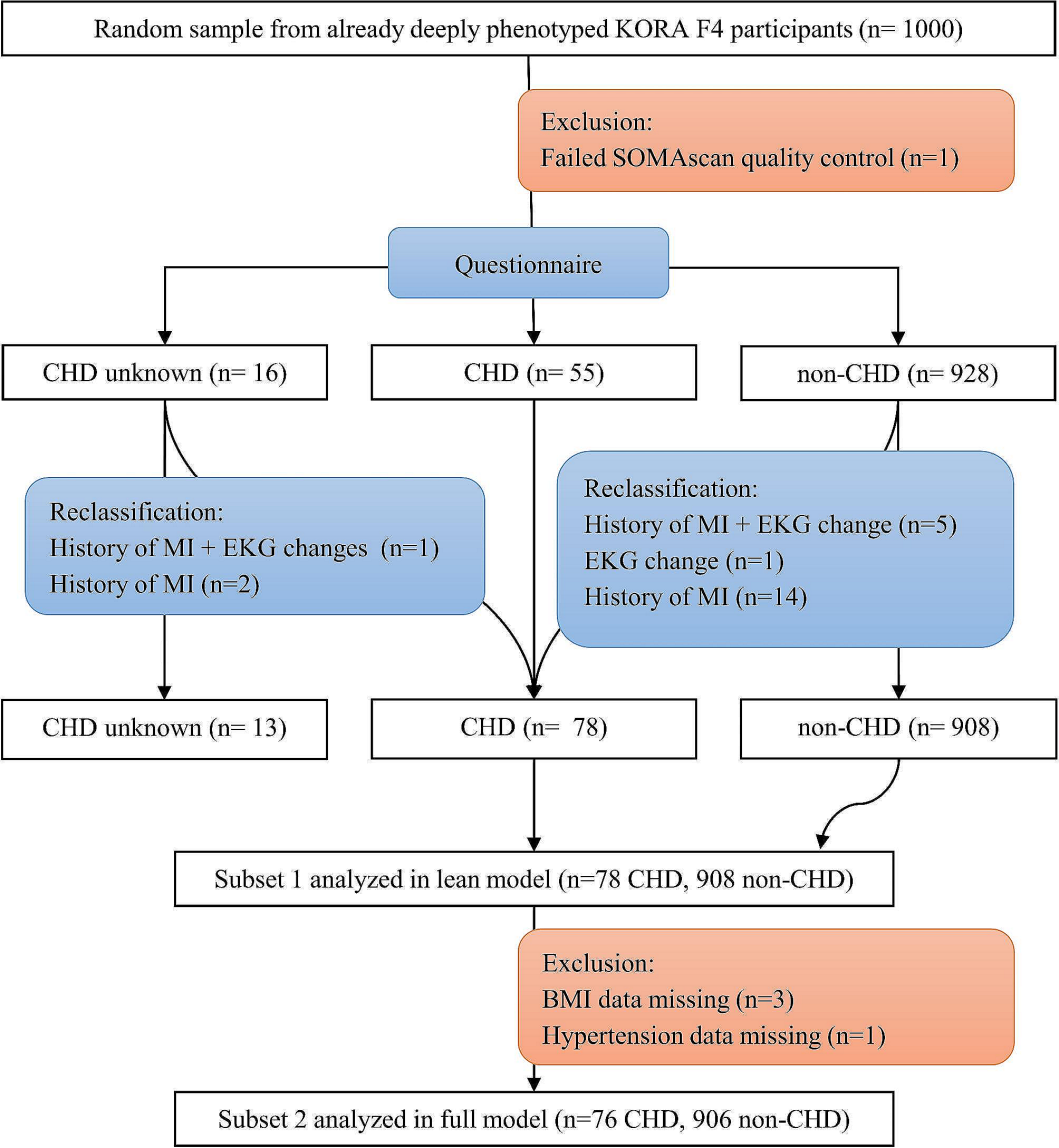
Flow diagram of subjects' stratification for CHD association. Coronary heart disease (CHD) was validated using self-reported history of myocardial infraction (MI) and characteristic electrocardiogram MI changes

We used data from the KORA F4 study (*n* = 3080), which is the follow-up of the KORA S4 survey that was conducted between 2006 and 2008. A subsample of 1000 individuals was randomly selected from the deeply phenotyped KORA F4 participants for plasma protein measurement using the SOMAscan aptamer-based proteomics platform [23]. We included all participants with data for the diagnosis of CHD according to Fig. 1. Exclusion criteria comprised (1) participants with missing data on the outcome or (2) participants with missing data on the model co-variates (BMI, hypertension status, diabetes status, levels of LDL, HDL, and triglycerides, smoking status, and physical activity).

### CHD status validation

Stratification of subjects for CHD association was performed according to Fig. 1. We first validated self-reported CHD using self-reported history of myocardial infarction (MI) and characteristic electrocardiogram (ECG) MI changes. The PC-based BioSys system (Hörmann Medizintechnik, Zwönitz, Germany) was used to record ECGs, which were manually assessed by two cardiologists. Infarction-characteristic changes in the ECG were identified by consensus. We used the validated CHD status as a binary variable in subsequent statistical analysis. According to the CHD-status validation, and the inclusion and exclusion criteria, 76 CHD-cases and 906 controls were carried forward to subsequent analysis.

### CIMT measurement

Ultrasound measurements of the extracranial carotid arteries have been performed in KORA participants as previously described [24, 25]. Briefly, all measurements were conducted by two sonographers according to a standardized protocol [26]. Optimal images of both common carotid arteries (CCA) were identified and stored. Then, CIMT was ascertained over a length of 10 mm beginning at 0–5 mm of the dilatation of the distal CCA using an automated edge detection reading system (Prowin software, Medical Technologies International, USA). The final CIMT value was calculated as the average of the measurements of three frozen images from both the left and right CCA. Measurements of inter-sonographer (*n* = 30 CIMT measurements) and inter-reader variations (*n* = 50 CIMT measurements) showed coefficients of variations of 1.9% and 3.0% and Spearman correlation coefficients of ≥ 0.89 (Supplementary information: Supplementary Fig. 1) [25].

### Assessment of model covariates

BMI was calculated by dividing each participant’s weight in kilograms by the square of the participant´s height. Participants were categorized as hypertensive if they had a blood pressure measurement (≥ 140/90 mmHg) or if they were receiving medical treatment for hypertension. Diabetes was defined by self-report or current use of glucose-lowering agents. Diabetes status was validated by asking the participant’s responsible physician. Lipid levels included low-density lipoprotein (LDL), high-density lipoprotein (HDL), and triglycerides. Self-reported smoking status was categorized as non-, former, or current smoker. Leisure time physical activity was assessed with two separate questions concerning leisure time sport activity in winter and in summer (cycling included). Possible answers were: (1) > 2 h, (2) 1–2 h, (3) < 1 h, and (4) none per week. Participants who had a total score < 5, obtained by summing the numbers (1)– (4) from the winter and summer questions, were classified as physically active.

### Proteomics measurements

Plasma samples were sent to SomaLogic Inc. (Boulder Colorado, USA) for proteomics measurement by means of the SOMAscan platform [23, 27]. Samples and bead coupled SOMAmers were mixed in solution. Free proteins were washed out and SOMAmer-protein complexes were photocleaved off the beads. Dextran sulfate was added to the solution as an anionic competitor, allowing non-cognate complexes to dissociate. SOMAmer-protein complexes were captured onto new avidin-coated beads by protein biotin tags. Free SOMAmers were washed out. Afterwards, SOMAmers were released from complexes in a denaturing buffer. Thus, individual protein concentrations were transformed into a corresponding SOMAmer concentration. SOMAmers were hybridized to complementary sequences on a microarray and quantified by fluorescence. The resulting raw intensities were processed using SOMALogic’s data analysis workflow, which utilizes the standard samples included on each plate and entails hybridization normalization, median signal normalization, and signal calibration to control for interplate differences. Protein intensities were reported in relative fluorescence units. One sample failed SOMAscan quality control, leaving a total of 999 samples from 483 males and 516 females. Additionally, we removed 29 aptamers that failed SOMAscan quality control, and 5 additional aptamers as recommended by the SOMAscan assay change log issued on December 22, 2016, leaving 1095 aptamers for further analysis. QC samples had a median intra-assay coefficient of variation (CV) of < 3% and a median inter-assay CV of < 7%. Proteomics data skipping median normalization were used to perform sensitivity analyses to investigate whether the quality control has an influence on the results.

### Study design of the replication cohort

For replication analysis, a matched CHD case-control sample was drawn from the KORA F3 cohort, which is a follow-up study from the previous KORA S3 survey, with enrolled German nationals between 25- and 74-years old living in the region of Augsburg, South Germany. The KORA F3 cohort consists of 3988 participants, whose data were collected between 2004 and 2005. 165 CHD-cases were drawn from the study as well as 165 (5-year interval) matched controls. Inclusion criteria for study participants comprised (1) biosample availability and (2) participants with data for the diagnosis of CHD according to the following criteria: self-reported CHD diagnosis, cardiac catheterization, bypass-operation, or myocardial infraction. Exclusion criteria comprised (1) participants with unkown status of CHD and (2) participants with missing data of the model co-variates (BMI, levels of LDL, HDL, triglycerides, diabetes status, hypertension status, smoking status, and physical activity).

To validate the results of our discovery phase, the replication was performed using ELISA-based measures of the significant proteins obtained with the fully-adjusted model. However, only galectin-4 was assessed to be associated with CHD. Due to the lack of CIMT measures in KORA F3, NCK1 was not tested.

### Enzyme-linked immunosorbent assay (ELISA) for galectin-4

An ELISA pre-coated with antibodies against galectin-4 was purchased from MyBiosource (San Diego, CA, U.S.A, MBS9715486) and analyzed according to the manufacturer’s protocol. A standard curve was prepared using recombinant galectin-4 in concentrations ranging from 96 to 3 pg/ml with a measurement error below 12%. Plasma samples were diluted in a 1:5 ratio using the dilution buffer provided by the kit. Absorption at 450 nm was measured with the Fluorostar Omega microplate reader (BMG LabTech, Ortenberg, Germany).

### Pre-processing of protein data

All protein values including either SOMAscan- or ELISA-based measures, were log2 transformed and standardized to have a mean of 0 and a standard deviation of 1, to be used for further statistical analysis.

### Statistical analysis

Estimated associations between CHD and each plasma protein were calculated by applying iterative multiple logistic regression analysis (dichotomous outcome), while associations between CIMT and each plasma protein were calculated using a linear regression model (continuous outcome). Both CHD and CIMT associations were tested according to the following models: model 1 adjusted by age and sex only; model 2 adjusted for age, sex, body mass index (BMI), smoking status, physical activity, low-density lipoprotein (LDL), high-density lipoprotein (HDL), triglyceride levels, diabetes status, and hypertension status.

Subjects with missing values were excluded from the respective analysis. To account for protein multiple testing, we applied Bonferroni correction resulting in a significant threshold of 4.57E-05 (0.05/1095 proteins) for both CHD and CIMT outcomes. To assess the performance of our replicated results as biomarkers, we calculated the area under the receiver operating characteristic curve (ROC-AUC) using the “pROC” R-package version 1.16.2.

### Systems biology analysis

Systems biology analysis of CHD- and CIMT-nominally associated proteins (*p* < 0.05) identified with the fully-adjusted model was performed using the Ingenuity Pathways Knowledge (Qiagen, Redwook City, CA), a database of biological interactions and processes spanning from molecular (proteins, genes) to organism (diseases) levels. Ingenuity Pathway Analysis (IPA) uses enrichment analysis-approaches to calculate the significance of observing a candidate protein/gene set within the context of biological systems. IPA calculates the p-value for enrichment or overlap between the test set and the IPA knowledge base using Fisher´s Exact test. Significant activation was considered at z-score > 2 and significant inhibition at z-score < − 2. Pathway analyzes included causal networks and identification of upstream regulators.

## Results

Baseline characteristics of the study population are described in Table 1. Stratification of participants in our discovery cohort KORA F4 for CHD association is displayed in Fig. 1. There were 982 individuals included in the fully-adjusted model of which 76 had CHD. Non-CHD participants were younger with a mean age of 58.7 years and comprised more women (53.2%), compared to participants with CHD who had a mean age of 64.8 years and comprised less women (31.6%). Participants with CHD had a higher mean of CIMT (0.91) as compared to those without (0.87, p-value: 0.017). As expected, participants with CHD showed higher consumption rates of statins, platelet aggregation inhibitors, aspirin, antihypertensive medication, beta-blockers, and cardiac glycosides. However, anti-diabetic medication showed no significant differences.

**Fig. 2:**
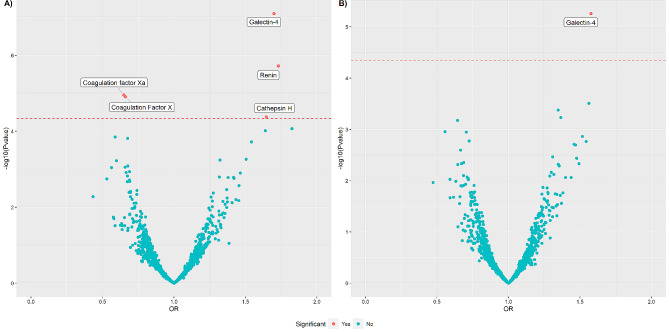
Volcano plot showing the association between plasma proteins and CHD. (**A**) Age-sex adjusted model. (**B**) Fully-adjusted model: age, sex, body mass index (BMI), physical activity, low-density lipoprotein (LDL), high-density lipoprotein (HDL), triglyceride levels, diabetes status, hypertension status and smoking status included as covariates. Each dot on the figure represents the odds ratio of a single protein with Bonferroni significant proteins labelled. OR: odds ratio

In total, five proteins were found to be significantly associated with CHD in the sex-age adjusted model (Table 2; Fig. 2,  Supplementary Table 1). Two of these proteins showed a protective effect namely, coagulation factor X (OR = 0.66, 95% CI = 0.55–0.80) and its activated form coagulation factor Xa (OR = 0.65, 95% CI = 0.53–0.79) and three showed negative effects namely, cathepsin H (OR = 1.64, 95% CI = 1.3–2.09), galectin-4 (OR = 1.70, 95% CI = 1.41–2.08) and renin (OR = 1.73, 95% CI = 1.38–2.17). Only galectin-4 remained significantly associated with CHD in the fully-adjusted model (OR = 1.58, 95% CI = 1.30–1.93) (Table 2; Fig. 2,  Supplementary Table 2)

**Table 1:** Baseline characteristics of the KORA F4 study sample: discovery phase

	Model 1: Age-sex adjusted model			Model 2: Fully-adjusted model		
	CHD		*P*-value	CHD		*P*-value
	No	Yes		No	Yes	
n	908	78		906	76	
Age (mean (SD))	58.74 (7.69)	64.72 (6.91)	< 0.001	58.74 (7.69)	64.75 (6.82)	< 0.001
Sex = Female (%)	483 (53.2)	24 (30.8)	< 0.001	482 (53.2)	24 (31.6)	< 0.001
BMI (mean (SD))	27.53 (4.46)	30.23 (5.27)	< 0.001	27.54 (4.46)	30.23 (5.27)	< 0.001
LDL (mean (SD))	140.56 (34.34)	127.60 (33.16)	0.001	140.69 (34.27)	127.95 (33.36)	0.002
HDL (mean (SD))	57.73 (15.11)	52.53 (15.82)	0.004	57.71 (15.12)	52.50 (15.83)	0.004
Triglycerides (mean (SD))	127.04 (88.49)	149.96 (72.50)	0.026	127.20 (88.52)	148.51 (72.89)	0.041
*Smoking status* (%)			0.013			0.013
Non-smoker	391 (43.1)	27 (34.6)		391 (43.2)	26 (34.2)	
Former smoker	375 (41.3)	45 (57.7)		373 (41.2)	44 (57.9)	
Current smoker	142 (15.6)	6 (7.7)		142 (15.7)	6 (7.9)	
Physically active = yes (%)	572 (63.0)	43 (55.1)	0.210	571 (63.0)	43 (56.6)	0.321
Diabetes status = yes (%)	67 (7.4)	14 (17.9)	0.002	67 (7.4)	14 (18.4)	0.002
Hypertension_status = yes (%)	334 (36.8)	54 (69.2)	< 0.001	334 (36.9)	53 (69.7)	< 0.001
Statins = yes (%)	91 (10.0)	45 (57.7)	< 0.001	91 (10.0)	43 (56.6)	< 0.001
Platelet aggregation inhibitors = yes (%)	60 (6.6)	45 (57.7)	< 0.001	60 (6.6)	45 (59.2)	< 0.001
Nitrates = yes (%)	1 (0.1)	1 (1.3)	0.370	1 (0.1)	1 (1.3)	0.360
Aspirin = yes (%)	81 (8.9)	45 (57.7)	< 0.001	81 (8.9)	45 (59.2)	< 0.001
Antihypertensive medication = yes (%)	246 (27.1)	63 (80.8)	< 0.001	246 (27.2)	61 (80.3)	< 0.001
Beta-blockers = yes (%)	138 (15.2)	54 (69.2)	< 0.001	138 (15.2)	52 (68.4)	< 0.001
Antidiabetic medication = yes (%)	47 (5.2)	6 (7.7)	0.494	47 (5.2)	6 (7.9)	0.460
Cardiac glycosides = yes (%)	1 (0.1)	3 (3.8)	< 0.001	1 (0.1)	3 (3.9)	< 0.001

CHD: coronary heart disease; BMI: body mass index; HDL: high-density lipoprotein; LDL: low-density lipoprotein; CIMT: carotid intima-media thickness; SD: standard deviation

**Table 2:** Associations between CHD and plasma proteins in both the discovery and replication phases of the study

a) Discovery phase: SOMAScan measurements					
Model 1: Age-sex adjusted model* KORA F4 sample: *n* = 78 CHD vs. 908 non-CHD					
Protein	UniProt	Gene symbol	OR	CI	*P*-value
Galectin-4	P56470	LGALS4	1.70	1.41–2.08	7.93E-08
Renin	P00797	REN	1.73	1.38–2.17	1.91E-06
Coagulation factor Xa	P00742	F10	0.65	0.53–0.79	1.10E-05
Coagulation Factor X	P00742	F10	0.66	0.55–0.80	1.23E-05
Cathepsin H	P09668	CTSH	1.64	1.30–2.09	4.21E-05

Model 2: Fully-adjusted model** KORA F4 sample: *n* = 76 CHD vs. 906 non-CHD					
Protein	UniProt	Gene Symbol	OR	CI	*P*-value
Galectin-4	P56470	LGALS4	1.58	1.30–1.93	5.50E-06

b) Replication phase: ELISA-based measures					
Model 2: Fully-adjusted model*** KORA F3 sample: *n* = 165 CHD vs. 165 non-CHD					
Protein	UniProt	Gene Symbol	OR	CI	*P*-value
Galectin-4	P56470	LGALS4	1.40	1.09–1.88	1.37E-02

*Model 1: Results from 1095 assessed proteins with the SOMAscan platform

**Model 2: Full model adjusted by age, sex, body mass index (BMI), low density lipoprotein (LDL), high density lipoprotein (HDL), triglyceride levels, diabetes status, hypertension status, smoking status (categorized as never, former or current smoker) and physical activity. Results from 1095 assessed proteins with the SOMAscan platform

***Results from testing galectin-4 only

CI: Confidence interval. OR: Odds ratio

**Fig. 3:**
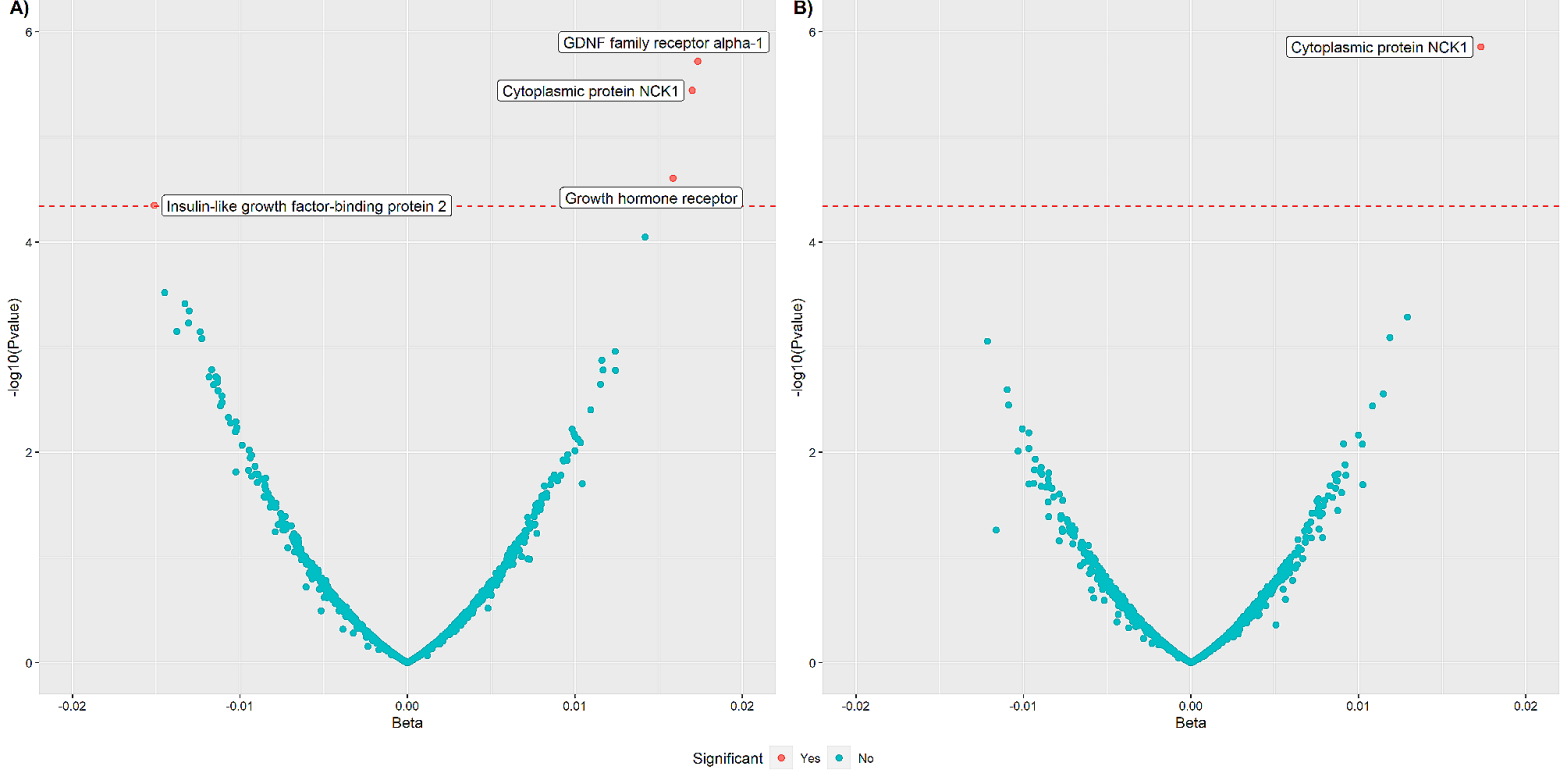
Volcano plot showing the association between plasma proteins and CIMT. (**A**) Age-sex adjusted model. (**B**) Fully-adjusted model: age, sex, body mass index (BMI), physical activity, low-density lipoprotein (LDL), high-density lipoprotein (HDL), triglyceride levels, diabetes status, hypertension status and smoking status included as covariates. Each dot on the figure represents the β-coefficients of a single protein with Bonferroni significant proteins labelled

For CIMT, four proteins in total were found to be significantly associated in the age-sex adjusted model after Bonferroni adjustment, these were GDNF family receptor alpha-1 (GFRA1) (β = 0.017, p-value = 1.91E-06), cytoplasmic protein NCK1 (NCK1) (β = 0.017, p-value = 3.59E-06), insulin-like growth factor-binding protein 2 (IGFBP2) (β = − 0.015, p-value = 4.47E-05), and growth hormone receptor (GHR) (β = 0.016, p-value = 2.46E-05) (Table 3; Fig. 3, Supplementary Table 3). In the fully-adjusted model, only NCK1 (β = 0.017, p-value = 1.39E-06) remained significantly associated with CIMT after Bonferroni adjustment (Table 3; Fig. 3, Supplementary Table 4).

**Table 3:** Associations between CIMT and plasma proteins in the discovery phase of the study

a) Discovery phase: SOMAScan measurements					
Model 1: Age-sex adjusted model* KORA F4 sample: *n* = 893					
Protein	UniProt	Gene symbol	Beta	SE	*P*-value
GDNF family receptor alpha-1	P56159	GFRA1	0.017	3.61E-03	1.91E-06
Cytoplasmic protein NCK1	P16333	NCK1	0.017	3.65E-03	3.59E-06
Growth hormone receptor	P10912	GHR	0.016	3.73E-03	2.46E-05
Insulin-like growth factor-binding protein 2	P18065	IGFBP2	-0.015	3.69E-03	4.47E-05

Model 2: Fully-adjusted model** KORA F4 sample: *n* = 889					
Protein	UniProt	Gene symbol	Beta	SE	*P*-value
Cytoplasmic protein NCK1	P16333	NCK1	0.017	3.56E-03	1.39E-06

*Model 1: Results from 1095 assessed proteins with the SOMAscan platform

**Model 2: Full model adjusted by age, sex, body mass index (BMI), physical activity, low density lipoprotein (LDL), high density lipoprotein (HDL), triglyceride levels, diabetes status, hypertension status and smoking status (categorized s never, former or current smoker). Results from 1095 assessed proteins with the SOMAscan platform

SE: Standard error

To investigate possible effects of the quality control of the proteomics data on the results [28], we performed a sensitivity analysis based on proteomics data skipping median normalization. No effect on our main CHD or CIMT findings was detected (Supplementary results: Sensitivity analyses; Supplementary Tables 6–7).

To validate our results, we tested our fully-adjusted model findings in an independent cohort of KORA F3 using ELISA-measured levels of galectin-4. Characteristics of participants are shown in Supplementary Table 5. The association between galectin-4 and CHD was successfully replicated in the fully-adjusted case-control study (OR = 1.40, 95% CI = 1.09–1.88, p-value = 1.37E-02; Supplementary Table 5).

To test galectin-4 as a biomarker, we run ROC-AUC tests in both KORA F3 and F4. A potential role of galectin-4 as a biomarker can be inferred from our results (Supplementary results: Assessment of Galectin-4 as a biomarker of CHD; Supplementary Figs. 2–3).

**Fig. 4:**
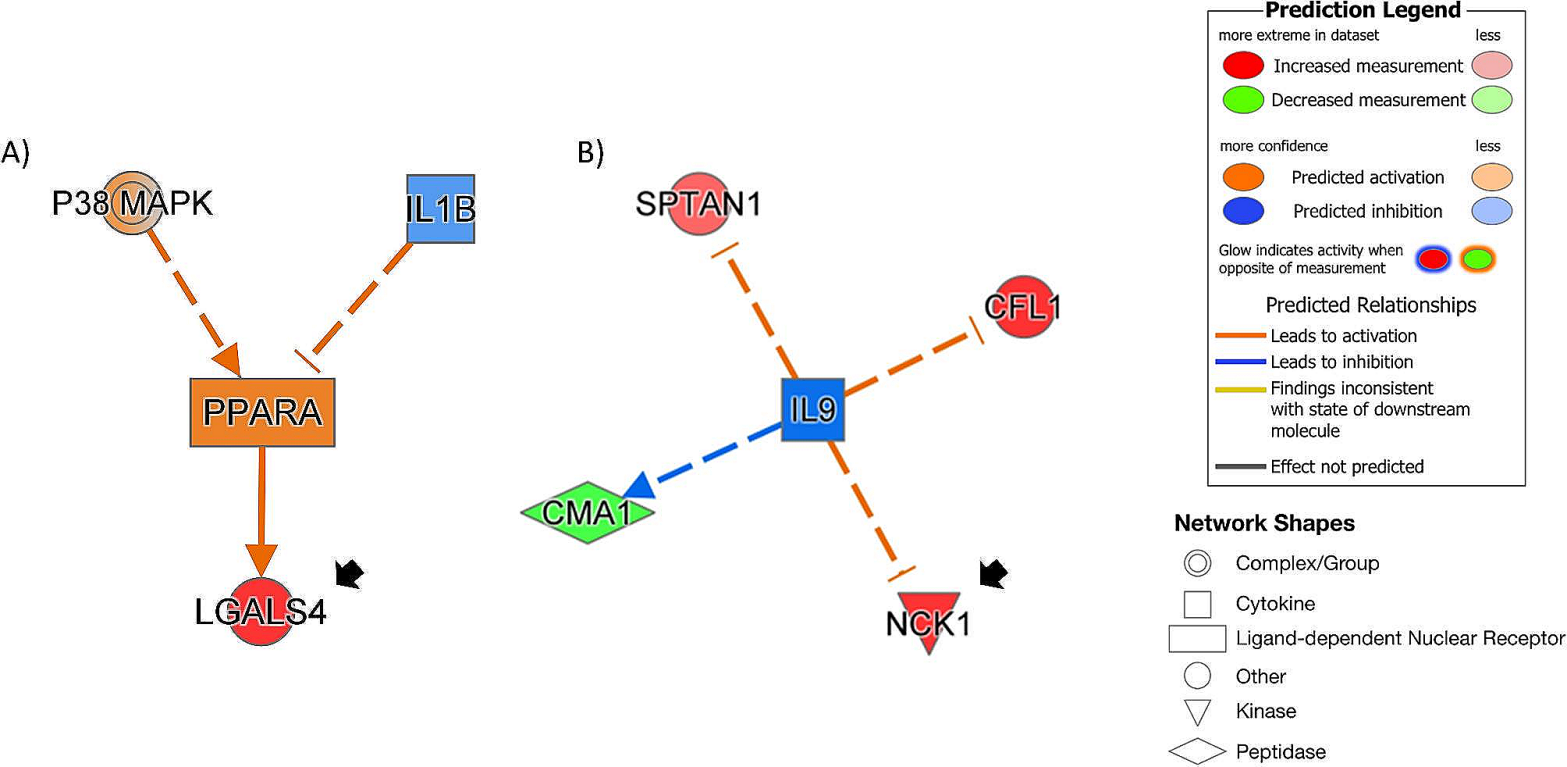
Predicted upstream regulators of galectin-4 or NCK1 and associated proteins. Results obtained when analyzing nominally significant (*p* < 0.05) CHD-associated proteins using the fully-adjusted model. (**A**) PPARA was predicted to directly increase expression of galectin-4 (LGALS4): selected pathways of the two significant causal networks predicting the activation of peroxisome proliferator activated receptor alpha (PPARA). Activation of P38 mitogen-activated protein kinase (P38MAPK) leads to activation and phosphorylation of PPARA. Interleukin-1 beta (IL1B) usually decreases the activity of PPARA; however, IL1B is predicted to be inhibited. (**B**) Unique significant inhibited causal network including NCK1: predicted inhibition of interleukin-9 (IL9) could indirectly increase expression of NCK1, SPTAN1 and CFL1, and indirectly decrease expression of CMA1. Full line: direct interaction; segmented line: indirect interaction. Black arrows: associated proteins identified by the fully-adjusted model in the discovery phase

Finally, to gain insights into enriched signaling pathways and biological mechanisms, Ingenuity Pathway Analysis was performed using nominally associated proteins (p-value < 0.05) identified with the fully-adjusted model including 106 CHD-associated proteins or 66 CIMT-associated proteins. For CHD, the unique significant causal network (z-score = − 2.11) including galectin-4 and 43 CHD-associated proteins, predicted the activation of peroxisome proliferator activated receptor alpha (PPARA), which might directly increase the expression of galectin-4. This finding was also predicted by the causal network with the lowest p-value (FDR = 5.37E-02), which included 68 CHD-associated proteins. Figure 4A shows the summary of PPARA-predicted activation via the two networks. For CIMT, the unique significant causal network (z-score = − 2.0) including NCK1 and four CIMT-associated proteins, identified interleukin-9 as an inhibited upstream regulator (Fig. 4B). Due to the prediction of inflammation pathways as upstream regulators of galectin-4 and NCK1, we further run the two models adjusting for high sensitivity C-reactive protein (CRP) in KORA F4 as a sensitivity analysis (Supplementary results: Sensitivity analyses; Supplementary Tables 8–9). No effect was observed on our main CHD or CIMT findings.

## Discussion

The main aim of the present study was to identify protein associations with CHD and CIMT, to provide understanding of the pathophysiology of CHD and atherosclerosis risk. In the age-sex adjusted model, proteomic analysis showed differential abundance of five proteins with CHD and four with CIMT. Following further adjustment of the model for BMI, smoking status, lipid measurements, hypertension, and diabetes status, the quantitative difference of galectin-4 for CHD and NCK1 for CIMT remained significant. Moreover, the association of galectin-4 with CHD was further validated using ELISA-based measurements in an independent study.

Galectin-4 is a member of the beta-galactoside-binding proteins, and has important functions in lipid raft stabilization, protein apical trafficking, cell adhesion, as well as wound healing [29]. Galectin-4 may be involved in atherosclerosis by enhancing lipid raft stabilization, which may subsequently affect redox signaling pathways [30]. Schroder et al. reported galectin-4 to be correlated with myocardial blood flow reserve, a gold standard diagnostic to clinically assess coronary microvascular dysfunction, in women with angina pectoris and non-obstructive CHD [31]. The authors conjectured that galectin-4’s promotion of cell adhesion contributed to the association [31]. A Swedish population-based study found galectin-4 to be significantly associated with incident coronary events (hazard ratio (HR) = 1.34, 95% confidence interval (CI) = 1.14–1.57) and incident heart failure (HR = 1.26, 95% CI 1.03–1.54) [32]. Another study compared heart failure patients to controls both recruited in the outpatient clinic at Karolinska University Hospital, finding galectin-4 to be significantly associated with heart failure (HR = 2.6; FDR adjusted p-value 0.005) [33]. In addition, galectin-4 has been reported to be associated with hospitalization linked to obesity [34] and ST-segment elevation myocardial infarction [35]. All listed reports are in line with our finding of galectin-4’s association with CHD. The pathway analysis of CHD-associated proteins suggests that the interplay of galectin-4 and the predicted activated status of both p38 MAPk signaling and interleukin-1B, representatives of inflammation pathways [36], takes place via the peroxisome proliferator activated receptor alpha (PPARA). PPARG-deficient macrophages have been found to display an elevated production of pro-inflammatory cytokines including interleukin-1B [37].

Among the five CHD-associated proteins using the age-sex adjusted model, two were found to be associated with a higher- and two with a lower-CHD risk. Our reported association of renin with higher CHD-risk might have been lost when using the fully-adjusted model due to the adjustment for hypertension [38]. However, renin has been reported to be positively associated with CHD [39, 40]. Renin is a member of the renin-angiotensin-aldosterone system, which, via its active peptide angiotensin II, contributes to atherosclerosis development, not only by promoting hypertension but also through multiple direct actions on vessels [41].

Cathepsin H was an additional protein associated with a higher CHD-risk in the age-sex adjusted model. Cathepsin H is a lysosomal cysteine protease important in the overall degradation of lysosomal proteins [42]. Its atherogenic role could lead the transformation of LDL to an atherogenic moiety, which in turn induces macrophage foam cell formation [43].

Proteins associated with lower-CHD risk associations included coagulation factor X as well as its active form coagulation factor Xa. A pathogenetic mechanism of CHD includes thrombotic vessel occlusion followed by rupture of an atherosclerotic plaque [3]. It is therefore not surprising that constituents and a regulating protein of the coagulation cascade were found significantly associated with CHD in the present study. Factor Xa exerts also non-hemostatic effects by activation of protease-activated receptors-1 (PAR-1) and PAR-2, which have been associated with atherosclerosis, inflammation, and fibrosis [44]. Such counterintuitive associations were reported before: where the concentrations of coagulation factor X and prothrombin were lower in blood from patients with CHD having more than 50% stenosis compared with those without CHD [45]. Brummel-Ziedins et al. hypothesized that despite the depletion of coagulation factors, the balance between tissue factor and tissue factor pathway inhibitor is the primary driver of a hypercoagulable state in patients with CHD [45].

We also identified proteins to be associated with CIMT. NCK1 was the unique significant protein positively associated with CIMT in the fully-adjusted model. NCK1 is reported to be involved in different pathways leading to the progression of atherosclerosis [46]. For instance, it is associated with vascular permeability, which allows the uptake of low-density lipoproteins (LDL) and thereby stimulates inflammation [46, 47]. The resulting oxidative stress increase in endothelial cells decreases nitric oxide and thereby supports endothelial cell dysfunction [48]. Alfaidi et al. showed in an in-vivo study that NCK1-knockdown reduced NF-κB signaling and thereby inflammation in endothelial cells [46]. Additionally, pathway analysis revealed interleukin-9 to indirectly inhibit expression of NCK1, SPTAN1, and CFL1, while the opposite effect was predicted for CMA1. Interleukin-9 has been reported to decrease expression of human NCK1 in MDA-MB-231 human breast cells, and to differentially regulate actin cytoskeleton-related proteins such as NCK1 [49]. Interestingly, both SPTAN1 and CFL1 are filamentous cytoskeletal and actin-related proteins, while CMA1 could participate in extracellular matrix degradation [50]. We were unable to validate the NCK1 findings using ELISA-measurements due to the lack of CIMT measurements in the validation sample.

We were able to confirm three additional proteins associated with CIMT using our age-sex adjusted model namely GFRA1, IGFBP2 and GHR, which have been all reported to be linked to CVD, mortality, and atherosclerosis [51, 52]. IGFBP2 was the unique obtained negative association. The same direction effect has been reported as a strong association with type 2 diabetes in a comparison study of incident type 2 diabetes and coronary heart disease in the KORA cohort [53].

In this study, we identified proteins specifically associated with either CHD or CIMT. Previous reports on KORA F4 have already stated a non-linear relation between CIMT and CHD risk [54]. Thus, this complex relationship amongst the two phenotypes could help to explain the specificity of our findings. In a sensitivity analysis additionally adjusting for CRP, the results of our full models did not change. Thus, our reported associations are independent from CRP, an inflammation marker of the acute phase response.

A major strength of our study is the proteome-wide approach, which covers proteins at low abundance levels in plasma. By conducting a hypothesis-free analysis, we were able to analyze the association of a wide array of plasma proteins with CHD and CIMT. An additional strength is the availability of an independent sample, which we could use to validate initial results with an alternative measurement technique that provides absolute concentrations. Our study also has limitations. The lack of patient differentiation by CHD severity could, for instance, be attenuating some associations. Manifestations of early-stage CHD differ from the late-stage CHD. In the latter, protein levels change due to myocardial injury and physiological compensation. Additionally, the difference in plaque vulnerability and extent of atherosclerosis between stable and unstable CHD [55], may also impact the plasma proteome. Finally, due to the cross-sectional nature of our study, temporal relations cannot be inferred.

In summary, our proteome-wide study identified a new association of galectin-4 with CHD. Galectin-4 may be involved in atherosclerosis by enhancing lipid raft stabilization, which subsequently affects redox signaling pathways. Moreover, we report the association of NCK1 with CIMT.

### Electronic supplementary material

Below is the link to the electronic supplementary material.

Supplementary Material 1

Supplementary Material 2

## Data Availability

The informed consent given by KORA study participants does not cover data posting in public databases. However, data are available upon request by means of a project agreement from KORA (https://helmholtz-muenchen.managed-otrs.com/external).
