# Supplementary material for: Plasma proteome association with coronary heart disease and carotid intima media thickness: results from the KORA F4 study
Source: Cardiovasc Diabetol. 2024 May 29;23:181. doi: 10.1186/s12933-024-02274-3 (PMC11138055; doi:10.1186/s12933-024-02274-3)
Supplement: Supplementary file 2 — Supplementary Material 2 [file 12933_2024_2274_MOESM2_ESM.docx]

**Plasma Proteome Association with Coronary Heart Disease and Carotid Intima Media Thickness: results from the KORA-F4 study**

Elhadad M et al.

**Supplementary information**

**Supplementary methods**


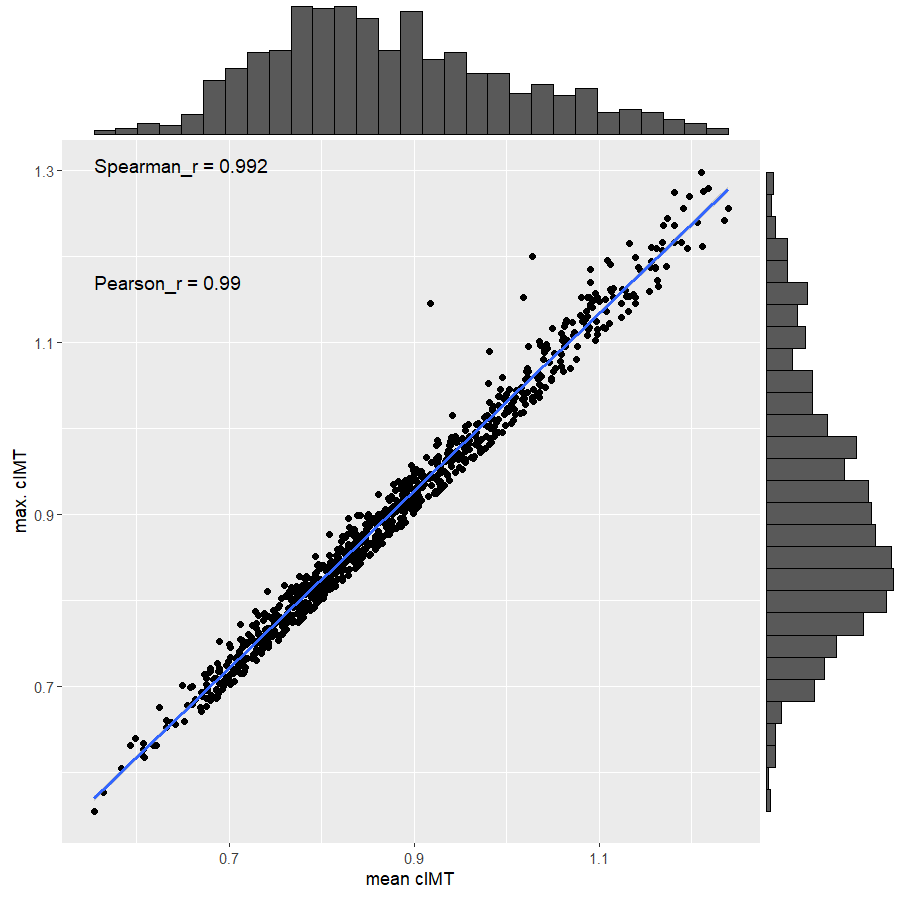


**Supplementary figure 1. Correlation between maximum and mean CIMT measurements.** Measurements of inter-sonographer (n = 30 CIMT measurements) and inter-reader variations (n  =  50 CIMT measurements) showed coefficients of variations of 1.9% and 3.0% and Spearman correlation coefficients of ≥ 0.89.

**Supplementary results**

*Sensitivity analyses*

A first sensitivity analysis to investigate whether the quality control of the data has an influence on the results included running our two models based on proteomics data skipping the median normalization (Supplementary tables 6 - 7). Results with the age-sex adjusted model resulted in 10 additional hits associated with CHD and two with CIMT. Results from the full model remained unchanged. Thus, quality control of the data does not have an influence on our main findings.

**Supplementary Table 6: Sensitivity analysis - Significant results of associations between CHD and plasma proteins with the two models based on data skipping the median normalization of the data.**

| **Model 1: Age-sex adjusted model***  **KORA F4 sample: n = 78 CHD vs. 908 non-CHD** | | | | | |
| --- | --- | --- | --- | --- | --- |
| **Protein** | **UniProt** | **Gene Symbol** | **OR** | **CI** | **P-value** |
| Leptin^ß^ | P41159 | LEP | 1.90 | 1.42 - 2.58 | 2.15E-05 |
| Angiopoietin-2^ß^ | O15123 | ANGPT2 | 1.65 | 1.32 - 2.07 | 1.48E-05 |
| Matrilysin^ß^ | P09237 | MMP7 | 1.66 | 1.32 - 2.11 | 2.07E-05 |
| Insulin-like growth factor-binding protein 4^ß^ | P22692 | IGFBP4 | 1.85 | 1.44 - 2.42 | 3.03E-06 |
| Galectin-4 | P56470 | LGALS4 | 1.80 | 1.48 - 2.21 | 9.85E-09 |
| Tumor necrosis factor ligand superfamily member 13B^ß^ | Q9Y275 | TNFSF13B | 1.56 | 1.27 - 1.90 | 1.46E-05 |
| Renin | P00797 | REN | 1.81 | 1.45 - 2.27 | 2.04E-07 |
| Cathepsin H | P09668 | CTSH | 1.94 | 1.52 - 2.47 | 7.99E-08 |
| Proteasome subunit alpha type-2^ß^ | P25787 | PSMA2 | 1.42 | 1.2 - 1.69 | 3.19E-05 |
| Roundabout homolog 2^ß^ | Q9HCK4 | ROBO2 | 1.90 | 1.46 - 2.51 | 3.36E-06 |
| Proprotein convertase subtilisin/kexin type 9^ß^ | Q8NBP7 | PCSK9 | 1.62 | 1.29 - 2.04 | 2.68E-05 |
| CD166 antigen^ß^ | Q13740 | ALCAM | 1.87 | 1.44 - 2.44 | 2.53E-06 |
| Testican-2^ß^ | Q92563 | SPOCK2 | 1.59 | 1.28 - 1.98 | 3.25E-05 |
| **Model 2: Fully-adjusted model****  **KORA F4 sample: n=76 CHD vs. 906 non-CHD** | | | | | |
| **Protein** | **UniProt** | **Gene Symbol** | **OR** | **CI** | **P-value** |
| Galectin-4 | P56470 | LGALS4 | 1.67 | 1.33 - 2.08 | 4.54E-06 |

*Model 1: Model adjusted by age and sex only.

**Model 2: Full model adjusted by age, sex, body mass index (BMI), low density lipoprotein (LDL), high density lipoprotein (HDL), triglyceride levels, diabetes status, hypertension status, smoking status (categorized as never, former or current smoker) and physical activity.

^ß^ Specific results of the sensitivity analysis.

CI: Confidence interval. OR: Odds ratio.

**Supplementary Table 7: Sensitivity analysis - Significant results of associations between CIMT and plasma proteins with the two models based on data skipping the median normalization of the data.**

| **Model 1: Age-sex adjusted model***  **KORA F4 sample: n = 893** | | | | | |
| --- | --- | --- | --- | --- | --- |
| **Protein** | **UniProt** | **Gene Symbol** | **Beta** | **SE** | **P-value** |
| Growth hormone receptor | P10912 | GHR | 0.017 | 0.004 | 5.67E-06 |
| GDNF family receptor alpha-1 | P56159 | GFRA1 | 0.018 | 0.004 | 1.59E-06 |
| Complement factor H^ß^ | P08603 | CFH | 0.016 | 0.004 | 4.91E-06 |
| Platelet-activating factor acetylhydrolase^ß^ | Q13093 | PLA2G7 | 0.016 | 0.004 | 1.43E-05 |
| Cytoplasmic protein NCK1 | P16333 | NCK1 | 0.018 | 0.004 | 6.35E-07 |
| **Model 2: Fully-adjusted model****  **KORA F4 sample: n = 889** | | | | | |
| **Protein** | **UniProt** | **Gene Symbol** | **Beta** | **SE** | **P-value** |
| Cytoplasmic protein NCK1 | P16333 | NCK1 | 0.016 | 0.004 | 1.03E-05 |

*Model 1: Model adjusted by age and sex only.

**Model 2: Full model adjusted by age, sex, body mass index (BMI), low density lipoprotein (LDL), high density lipoprotein (HDL), triglyceride levels, diabetes status, hypertension status, smoking status (categorized as never, former or current smoker) and physical activity.

^ß^ Specific results of the sensitivity analysis.

A second sensitivity analysis was performed to investigate whether the C-reactive protein (CRP) – a marker of acute inflammation –, has an influence on the results of our two models (Supplementary tables 8 - 9). For the sex-age model, association of CHD with cathepsin H, and association of CIMT with both growth hormone receptor and IGFBP2 were lost after adjusting for CRP. None of the associations with CHD or CIMT was changed when using the fully-adjusted model.

**Supplementary Table 8: Sensitivity analysis - Significant results of associations between CHD and plasma proteins with the two models with additional adjustment for CRP.**

| **Model 1: Age-sex adjusted model + CRP***  **KORA F4 sample: n = 78 CHD vs. 908 non-CHD** | | | | | |
| --- | --- | --- | --- | --- | --- |
| **Protein** | **UniProt** | **Gene Symbol** | **OR** | **CI** | **P-value** |
| Coagulation Factor X | P00742 | F10 | 0.67 | 0.55 - 0.80 | 2.16E-05 |
| Coagulation factor Xa | P00742 | F10 | 0.65 | 0.54 - 0.79 | 1.73E-05 |
| Galectin-4 | P56470 | LGALS4 | 1.69 | 1.39 - 2.06 | 1.13E-07 |
| Renin | P00797 | REN | 1.72 | 1.37 - 2.16 | 2.24E-06 |
| **Model 2: Fully-adjusted model + CRP****  **KORA F4 sample: n=76 CHD vs. 906 non-CHD** | | | | | |
| **Protein** | **UniProt** | **Gene Symbol** | **OR** | **CI** | **P-value** |
| Galectin-4 | P56470 | LGALS4 | 1.58 | 1.29 - 1.93 | 5.46E-06 |

*Model 1: Model adjusted by age and sex only + CRP.

**Model 2: Full model adjusted by age, sex, body mass index (BMI), low density lipoprotein (LDL), high density lipoprotein (HDL), triglyceride levels, diabetes status, hypertension status, smoking status (categorized as never, former or current smoker) and physical activity + CRP.

CI: Confidence interval. OR: Odds ratio.

**Supplementary Table 9: Sensitivity analysis - Significant results of associations between CIMT and plasma proteins with the two models with additional adjustment for CRP.**

| **Model 1: Age-sex adjusted model + CRP***  **KORA F4 sample: n = 78 CHD vs. 908 non-CHD** | | | | | |
| --- | --- | --- | --- | --- | --- |
| **Protein** | **UniProt** | **Gene Symbol** | **Beta** | **SE** | **P-value** |
| GDNF family receptor alpha-1 | P56159 | GFRA1 | 0.02 | 0.003 | 8.21E-06 |
| Cytoplasmic protein NCK1 | P16333 | NCK1 | 0.02 | 0.004 | 3.55E-06 |
| **Model 2: Fully-adjusted model + CRP****  **KORA F4 sample: n=76 CHD vs. 906 non-CHD** | | | | | |
| **Protein** | **UniProt** | **Gene Symbol** | **Beta** | **SE** | **P-value** |
| Cytoplasmic protein NCK1 | P16333 | NCK1 | 0.02 | 0.003 | 1.42E-06 |

*Model 1: Model adjusted by age and sex only + CRP.

**Model 2: Full model adjusted by age, sex, body mass index (BMI), low density lipoprotein (LDL), high density lipoprotein (HDL), triglyceride levels, diabetes status, hypertension status, smoking status (categorized as never, former or current smoker) and physical activity + CRP.

CI: Confidence interval. OR: Odds ratio.

*Assessment of Galectin-4 as a biomarker of CHD*

We assessed the potential of galectin-4 as CHD biomarker by using area under the receiver operating characteristic curve (ROC-AUC). We tested galectin-4 as a predictor of CHD in KORA-F4 and validated the results in KORA-F3 (KORA-F4 AUC = 0.72, KORA-F3 AUC = 0.54) (Supplementary figure 2). Moreover, we assessed the performance of galectin-4 as a predictor of incident MI in KORA F4 (AUC = 0.67) (Supplementary figure 3). We acknowledge the limitations of our analyses including the lack of comparable validation cohort and the unavailability of data on incident CAD in our cohort. However, we opted to add these results in the supplement for interested readers. Additionally, our cohort lacked information on the disease severity of our cases, which might be relevant in the performance of the proteins as biomarkers.


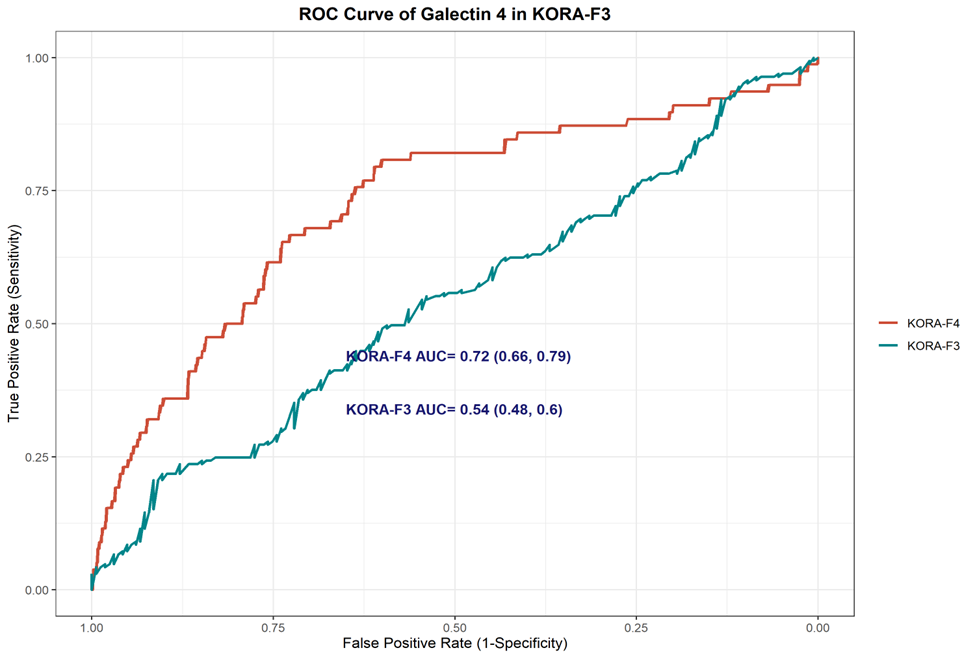


**Supplementary figure 2. Galectin-4 as predictor of CHD in KORA F4 and F3.** ROC curves for galectin-4 as predictor of CHD. In KORA F4 (n = 1000) the AUC-ROC value was 0.72, while the specificity and sensitivity values were 0.66 and 0.79, respectively. In KORA F3 (165 CAD cases and 165 controls) the AUC-ROC, specificity, and sensitivity values were 0.54, 0.48, and 0.6, respectively.


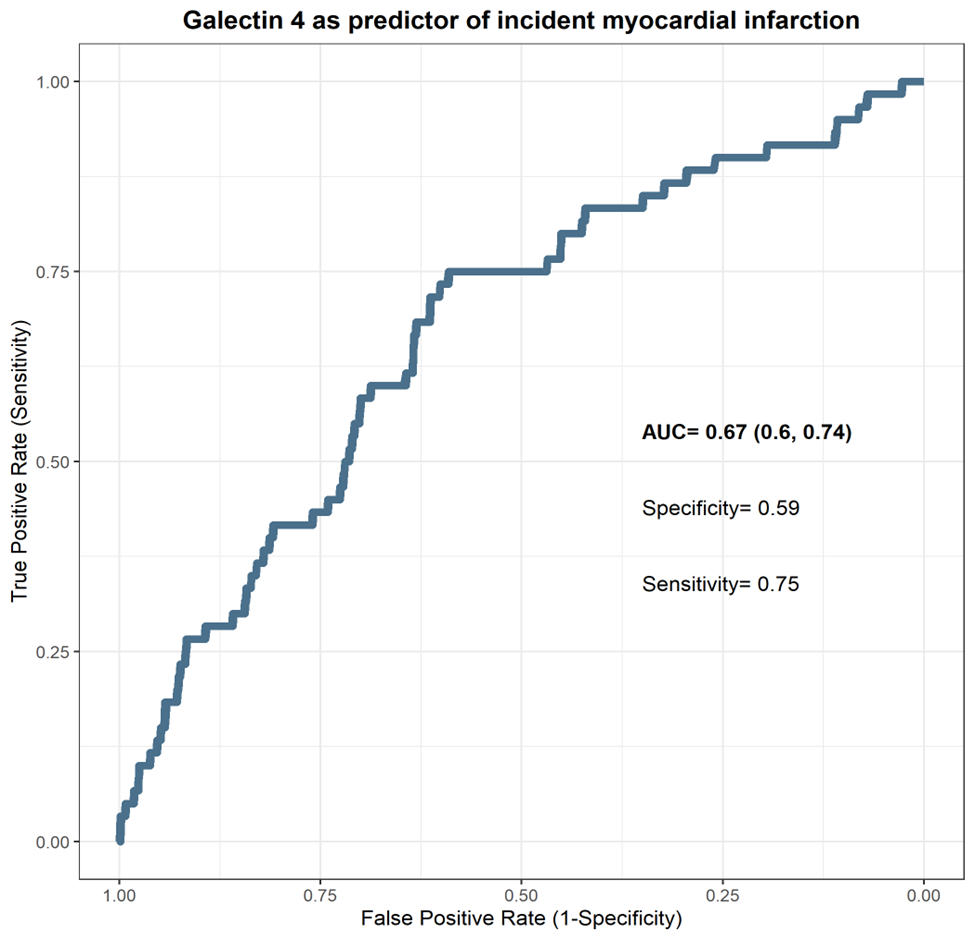


**Supplementary figure 3. Galectin-4 as predictor of incident MI in KORA F4.** A ROC-AUC value of 0.67 was obtained in KORA F4 (n = 1000). Specificity and sensitivity values were 0.59 and 0.75, respectively.

**Index of supplementary tables**

Supplementary table 1: Association between CHD and proteins using a sex-age adjusted model in KORA F4 (Excel file).

Supplementary table 2: Association between CHD and proteins using a fully adjusted model in KORA F4 (Excel file).

Supplementary table 3: Association between CIMT and proteins using a sex-age adjusted model KORA F4 (Excel file).

Supplementary table 4: Association between CIMT and proteins using a fully adjusted model KORA F4 (Excel file).

Supplementary table 5: KORA F3 case-control study: a) Characteristics of participants, b) Association of CHD and galectin 4 in KORA F3 (Excel file).

Supplementary table 6: Sensitivity analysis - Significant results of associations between CHD and plasma proteins with the two models based on data skipping the median normalization of the data

Supplementary table 7: Sensitivity analysis - Significant results of associations between CIMT and plasma proteins with the two models based on data skipping the median normalization of the data.

Supplementary Table 8: Sensitivity analysis - Significant results of associations between CHD and plasma proteins with the two models with additional adjustment for CRP.

Supplementary Table 9: Sensitivity analysis - Significant results of associations between CIMT and plasma proteins with the two models with additional adjustment for CRP.
